# Associations between Anticholinergic Burden and Adverse Health Outcomes in Parkinson Disease

**DOI:** 10.1371/journal.pone.0150621

**Published:** 2016-03-03

**Authors:** James A. G. Crispo, Allison W. Willis, Dylan P. Thibault, Yannick Fortin, Harlen D. Hays, Douglas S. McNair, Lise M. Bjerre, Dafna E. Kohen, Santiago Perez-Lloret, Donald R. Mattison, Daniel Krewski

**Affiliations:** 1 McLaughlin Centre for Population Health Risk Assessment, University of Ottawa, Ottawa, Ontario, Canada; 2 Fulbright Canada Student, University of Pennsylvania, Philadelphia, Pennsylvania, United States of America; 3 Department of Neurology, University of Pennsylvania Perelman School of Medicine, Philadelphia, Pennsylvania, United States of America; 4 Department of Biostatistics and Epidemiology, University of Pennsylvania Perelman School of Medicine, Philadelphia, Pennsylvania, United States of America; 5 Center for Clinical Epidemiology and Biostatistics, University of Pennsylvania Perelman School of Medicine, Philadelphia, Pennsylvania, United States of America; 6 Cerner Corporation, Kansas City, Missouri, United States of America; 7 C. T. Lamont Primary Health Care Research Centre, Department of Family Medicine, University of Ottawa, Ottawa, Ontario, Canada; 8 Bruyère Research Institute, Ottawa, Ontario, Canada; 9 School of Epidemiology, Public Health and Preventive Medicine, University of Ottawa, Ottawa, Ontario, Canada; 10 Cardiology Research Institute, University of Buenos Aires, National Research Council (ININCA-UBA-CONICET), Buenos Aires, Argentina; 11 Risk Sciences International, Ottawa, Ontario, Canada; University of Brescia, ITALY

## Abstract

**Background:**

Elderly adults should avoid medications with anticholinergic effects since they may increase the risk of adverse events, including falls, delirium, and cognitive impairment. However, data on anticholinergic burden are limited in subpopulations, such as individuals with Parkinson disease (PD). The objective of this study was to determine whether anticholinergic burden was associated with adverse outcomes in a PD inpatient population.

**Methods:**

Using the Cerner Health Facts® database, we retrospectively examined anticholinergic medication use, diagnoses, and hospital revisits within a cohort of 16,302 PD inpatients admitted to a Cerner hospital between 2000 and 2011. Anticholinergic burden was computed using the Anticholinergic Risk Scale (ARS). Primary outcomes were associations between ARS score and diagnosis of fracture and delirium. Secondary outcomes included associations between ARS score and 30-day hospital revisits.

**Results:**

Many individuals (57.8%) were prescribed non-PD medications with moderate to very strong anticholinergic potential. Individuals with the greatest ARS score (≥4) were more likely to be diagnosed with fractures (adjusted odds ratio (AOR): 1.56, 95% CI: 1.29–1.88) and delirium (AOR: 1.61, 95% CI: 1.08–2.40) relative to those with no anticholinergic burden. Similarly, inpatients with the greatest ARS score were more likely to visit the emergency department (adjusted hazard ratio (AHR): 1.32, 95% CI: 1.10–1.58) and be readmitted (AHR: 1.16, 95% CI: 1.01–1.33) within 30-days of discharge.

**Conclusions:**

We found a positive association between increased anticholinergic burden and adverse outcomes among individuals with PD. Additional pharmacovigilance studies are needed to better understand risks associated with anticholinergic medication use in PD.

## Introduction

Anticholinergic medications belong to a class of drugs that block muscarinic receptors and are used to treat a wide range of indications that more frequently present in elderly populations, including urinary incontinence, hypertension, respiratory disorders, and depression [1, 2]. Studies have found anticholinergic burden, defined as the cumulative anticholinergic potential resulting from polypharmacy [3], to be a significant risk factor for falls and fractures [4–7], delirium [6, 8], and cognitive impairment in elderly populations [9, 10]. Anticholinergic burden is also associated with hospital readmission among older adults [7, 11–13], which may be preventable. Knowledge of adverse outcomes associated with medications having anticholinergic properties has contributed to the development of numerous scales to quantify anticholinergic burden [13–15]. Validation studies have consistently shown that a higher anticholinergic burden score on any scale increases the risk of experiencing adverse events [15].

Disease-related disruptions to central cholinergic pathways may cause individuals with PD who have elevated anticholinergic burden to be more vulnerable to adverse effects compared to individuals with PD who are treated with fewer or less potent anticholinergic medications [16, 17]. Levodopa, pramipexole, selegiline, entacapone, and amantadine have mild anticholinergic effects, but are essential medications in the treatment of PD [18]. Common cardiac, gastrointestinal, allergy, pain, and psychiatric medications have anticholinergic effects as well, but these medications generally have alternatives. In order to develop clinical guidelines that may reduce preventable adverse events in PD, basic information on anticholinergic burden and its impact, if any, on the health and care of individuals with PD is required.

To address this important data gap, we used electronic health records from the Cerner Health Facts® database to determine non-PD anticholinergic medication use and examine the relationship between anticholinergic burden and adverse outcomes in a PD inpatient population. Our primary objectives were to determine whether anticholinergic burden was associated with the diagnosis of clinical outcomes, specifically fracture and delirium. Anticholinergic burden has been demonstrated to increase healthcare utilization among elderly adults [11]; however, its impact on the care of individuals with PD remains unknown. Our secondary objectives were therefore to examine whether anticholinergic burden was associated with emergency department (ED) visit and inpatient readmission within 30-days of inpatient discharge.

## Methods

This study was approved by the Health Sciences and Science Research Ethics Board at the University of Ottawa, Ottawa, Ontario, Canada (H05-13-24). Informed consent was not required from individuals included in this study, as all health records were anonymized and de-identified prior to our analyses.

### Data Source

Data for this study was obtained from the Cerner Corporation’s (Kansas City, Missouri) Health Facts® database. First implemented in subscribing care centers in January 2000, Health Facts® is a time-stamped electronic health record that contains in-depth patient demographic, encounter, clinical, laboratory, pharmacy, and billing data. To date, Health Facts® contains encounter-level health information on over 47 million individuals who have received care at any of the more than 600 subscribing centers. Approximately 65% of all data in Health Facts® originate from academic medical centers. Most outpatient pharmacy data is missing in Health Facts®; therefore, the database in best suited for inpatient drug-association studies and health services research.

### Cohort and Index Encounter Selection

The study cohort was comprised of hospitalized individuals with PD between January 2000 and December 2011. To be eligible for cohort entry, individuals had to have: 1) a recorded diagnosis of PD (in any setting) according to the International Classification of Diseases, Ninth Revision (ICD-9: code 332 for PD, or code 332.0 for Paralysis Agitans) and 2) one or more inpatient encounters > 2 days at or after the time of PD diagnosis in which diagnoses were recorded and medications were dispensed. Eligible encounters were restricted to those > 2 days in order to more accurately approximate outpatient pharmacotherapy, since outpatient medications may not be dispensed by hospital pharmacies for shorter stays (such as day surgeries) or to individuals admitted for fatal events. Individuals were excluded from our study if 1) they had a diagnosis (in any setting) of secondary parkinsonism (ICD-9, code 332.1) or other degenerative diseases of the basal ganglia (ICD-9, code 333.0) or 2) their age was undocumented or less than 40 years at first PD diagnosis, thus reducing the number of individuals with atypical PD or cases of incorrectly diagnosed PD from the cohort. The earliest qualifying inpatient encounter was then selected as the index encounter for each individual from the eligible study cohort (n = 17,337). Since our secondary objectives focused on 30-day hospital revisits, individuals who died during their index encounter were excluded from the cohort (n = 512). Lastly, to accommodate adjustment for *a priori* defined covariates, we restricted the cohort to individuals with complete sex and race data (n = 16,302).

### Demographics, Care Setting Characteristics, and Comorbidity

Demographic data examined and reported from index encounters were age at admission, sex (female or male), and race (Caucasian, African American, Hispanic, Asian, or other). Age at admission was categorized into 10-year age strata from 40–49 to 90+ years. Care setting characteristics derived and reported from index encounters included location (urban or rural), teaching status (teaching or non-teaching), census region (Northeast, South, Midwest, or West), and number of beds (<6, 6–99, 100–199, 200–299, 300–499, 500+). Length of stay of each index encounter was categorized as 3–6, 7–30, or 31+ days. Comorbidity was assessed at the index encounters using enhanced ICD-9-CM coding algorithms for Elixhauser comorbidities [19]. A weighted comorbidity summary score was then calculated for each individual using data from their index encounter [20].

### Anticholinergic Exposure

The Anticholinergic Risk Scale (ARS), a validated and pharmacist-developed weighted list of frequently prescribed medications that have anticholinergic potential, was used to calculate anticholinergic burden [18]. To appraise anticholinergic burden, we first reconciled medications dispensed during index encounters and examined the prevalent use of each ARS drug. Each individual’s ARS score was calculated as the weighted sum of ARS drugs dispensed during their index encounter and classified as 0, 1, 2–3, or 4+. Since the Cerner Health Facts® database does not contain detailed outpatient pharmacy data, information on the use of over-the-counter medications, or prescription adherence data, the ARS score was solely derived using data on medications dispensed in inpatient settings. We made the assumption that medications that were prescribed in the inpatient setting and not marked as canceled or not dispensed were actually administered to the patient.

### Outcomes Data

Our primary outcomes were inpatient diagnosis of fracture and delirium, with secondary outcomes being 30-day ED visit and inpatient readmission. Outcomes were selected based on prior reports of associations between anticholinergic burden and clinical and health service utilization outcomes in large cohorts of elderly adults [4–10]. Primary outcomes were defined as a recorded primary or secondary ICD-9 diagnosis of fracture (800.x–829.x) and delirium (293.x), respectively, during the index encounter. As it is not possible to follow individual patients across different Health Facts® subscribing care centers, secondary outcomes were defined as ED visit or inpatient readmission at the same care center within 30-days of index encounter discharge. Prior to examining hospital revisits, we confirmed that all index encounter care centers were still active subscribers to Health Facts® 30-days post individual inpatient discharge. Thirty-day ED visit and inpatient readmission post index encounter discharge was then coded as a binary variable, with the minimum time to hospital return recorded for all events.

### Statistical Analyses

Descriptive statistics were used to report demographic, clinical, and care setting characteristic, as well as the prevalent use of individual ARS drugs. To determine the association between anticholinergic burden and inpatient diagnosis of adverse events (fracture or delirium), we constructed unconditional logistic regression models that computed unadjusted and adjusted odds of adverse event compared to a reference group (ARS score = 0, no anticholinergic burden) for each category of anticholinergic burden (ARS score = 1, 2–3, and 4+). For secondary outcomes, we calculated the risk of 30-day ER visit and inpatient readmission using a time-to-event analysis. Cox proportional hazard models were constructed to determine the unadjusted and adjusted risk of 30-day ED visit and inpatient readmission relative to a reference group (ARS score = 0) for each category of anticholinergic burden (ARS score = 1, 2–3, and 4+). Multivariable logistic regression and Cox proportional hazard models included the following demographic, clinical, and care setting characteristic that were hypothesized *a priori* to be potential confounders: age (continuous), sex, race, length of stay, comorbidity score, census region, urban/rural status, hospital size (number of beds), and hospital teaching status. All analyses were completed using SAS v9.4 (SAS Institute Inc., Cary, NC, USA).

## Results

### Cohort Characteristics

There were 16,302 individuals with PD who satisfied our inclusion criteria and were admitted to hospital between January 1, 2000 and December 31, 2011 (Table 1). Consistent with the demographics of other large PD populations, men (52.3%) comprised the majority of our study cohort and individuals were older (82.3% were 70 years of age or older) at admission [21, 22]. Individuals were predominantly Caucasian (91.2%), while others identified as African American (n = 1,061; 6.5%), Hispanic (1.0%), Asian (0.6%), or other (0.8%) races. Nearly all (99.1%) inpatient encounters were 30 days or less. Most study encounters took place at large (300+ beds, 46.8%), urban (99.8%), academic (64.1%), Northeast (49.0%) care centers.

**Table 1:** Demographic characteristics of study cohort.

Characteristic		Study Cohort n (%) (n = 16,302)
**Age**	** **	
	40–49	125 (0.8)
	50–59	630 (3.9)
	60–69	2,121 (13.0)
	70–79	5,486 (33.7)
	80–89	6,753 (41.4)
	90+	1,187 (7.3)
**Sex**	** **	
	Female	7,730 (47.4)
	Male	8,572 (52.6)
**Race**	** **	
	Caucasian	14,861 (91.2)
	African American	1,061 (6.5)
	Hispanic	164 (1.0)
	Asian	91 (0.6)
	Other	125 (0.8)
**Length of stay**	** **	
	3–6 days	10,362 (63.6)
	7–30 days	5,799 (35.6)
	31+ days	141 (0.9)
**ARS score**	** **	
	0	2,463 (15.1)
	1	4,280 (26.3)
	2–3	4,762 (29.2)
	4+	4,797 (29.4)
		
**Mean Comorbidity Score**^a^	** **	6.1 ± 6.7
**Payer status**	** **	
	Medicare	7,228 (44.3)
	Public	377 (2.3)
	Private	799 (4.9)
	Uninsured	258 (1.6)
	Missing	7,640 (46.9)
**Care setting location**	** **	
	Urban	16,266 (99.8)
	Rural	36 (0.2)
**Teaching status**	** **	
	Non-teaching	5,848 (35.9)
	Teaching	10,454 (64.1)
**Census region**	** **	
	Northeast	7,984 (49.0)
	South	4,007 (24.6)
	Midwest	3,220 (19.8)
	West	1,091 (6.7)
**Number of beds**	** **	
	<6	53 (0.3)
	6–99	1,193 (7.3)
	100–199	2,365 (14.5)
	200–299	5,065 (31.1)
	300–499	3,418 (21.0)
	500+	4,208 (25.8)

Abbreviation: ARS, anticholinergic risk scale.

^a^Mean ± standard deviation.

### Anticholinergic Exposure

The majority (57.8%) of individuals included in our study were prescribed one or more non-PD medications with anticholinergic effects. Any use of medications with anticholinergic properties was common among individuals in our study, with similar proportions of individuals in examined ARS score strata (ARS score 1: 26.3%; 2–3: 29.2%; 4+: 29.4%) (Table 1). Individuals were frequently prescribed one or more medications with a moderate anticholinergic potential (1 point, 77.1%), which were primarily antiparkinson agents (levodopa, 60.7%; pramipexole, 8.4%; entacapone, 6.3%; and selegiline, 2.5%) (Table 2). Medications with strong (2 points) and very strong (3 points) anticholinergic potential were prescribed to 19.9% and 25.4% of inpatients, respectively.

**Table 2:** Prevalent use of individual ARS medications by anticholinergic potential in study cohort.

3 Points	n (%)	2 Points	n (%)	1 Point	n (%)
**Any 3 point ARS drug**	4,135 (25.4)	**Any 2 point ARS drug**	3,252 (19.9)	**Any 1 point ARS drug**	12,570 (77.1)
diphenhydramine hydrochloride	1,241 (7.6)	olanzapine	750 (4.6)	carbidopa-levodopa^a^	9,900 (60.7)
promethazine hydrochloride	1,002 (6.1)	amantadine hydrochloride^a^	663 (4.1)	quetiapine fumarate	1,563 (9.6)
oxybutynin chloride	552 (3.4)	tolterodine tartrate	543 (3.3)	metoclopramide hydrochloride	1,426 (8.7)
atropine products	417 (2.6)	loratadine	348 (2.1)	pramipexole dihydrochloride^a^	1,374 (8.4)
hydroxyzine hydrochloride or hydroxyzine pamoate	391 (2.4)	prochlorperazine maleate	341 (2.1)	haloperidol	1,041 (6.4)
benztropine mesylate	358 (2.2)	loperamide hydrochloride	300 (1.8)	entacapone^a^	1,021 (6.3)
amitriptyline hydrochloride	284 (1.7)	cyclobenzaprine hydrochloride	211 (1.3)	risperidone	839 (5.1)
meclizine hydrochloride	245 (1.5)	baclofen	153 (0.9)	mirtazapine	799 (4.9)
hyoscyamine products	104 (0.6)	nortriptyline hydrochloride	92 (0.6)	paroxetine hydrochloride	714 (4.4)
dicyclomine hydrochloride	92 (0.6)	cetirizine hydrochloride	82 (0.5)	trazodone hydrochloride	669 (4.1)
tizanidine hydrochloride	53 (0.3)	clozapine	68 (0.4)	ranitidine hydrochloride	566 (3.5)
chlorpromazine hydrochloride	51 (0.3)	cimetidine	52 (0.3)	selegiline hydrochloride^a^	411 (2.5)
perphenazine	43 (0.3)	desipramine hydrochloride	9 (0.1)	ziprasidone hydrochloride	148 (0.9)
cyproheptadine hydrochloride	42 (0.3)	pseudoephedrine hydrochloride-triprolidine hydrochloride	1 (0.0)	methocarbamol	90 (0.6)
imipramine hydrochloride	37 (0.2)				
carisoprodol	33 (0.2)				
thioridazine hydrochloride	19 (0.1)				
chlorpheniramine maleate	16 (0.1)				
fluphenazine hydrochloride	16 (0.1)				
trifluoperazine hydrochloride	13 (0.1)				
thiothixene	12 (0.1)				

Abbreviation: ARS, anticholinergic risk scale.

^a^Antiparkinson medication.

### Inpatient Diagnosis of Fracture and Delirium

Associations between anticholinergic burden and inpatient diagnosis of fracture and delirium are shown in Table 3A and 3B, respectively. Unadjusted models demonstrated a significant association between each strata of anticholinergic burden and fracture diagnosis (compared to individuals with ARS score = 0), with the association being greatest for individuals with the highest ARS scores (odds ratio (OR): 1.66, 95% CI: 1.38–1.99). Individuals with ARS scores > 1 were significantly more likely to be diagnosed with delirium compared to those prescribed medications without anticholinergic effects. Adjustment for relevant covariates, including potential confounders, weakened the observed associations only slightly. Results revealed that individuals with the highest ARS scores (≥4) had the greatest statistically significant risk of fracture (adjusted odds ratio (AOR): 1.56, 95% CI: 1.29–1.88) and that individuals with high to very high ARS scores were at significant risk of delirium compared to individuals with no anticholinergic burden (ARS score 2–3: AOR: 2.14, 95% CI: 1.46–3.15; 4+: AOR: 1.61, 95% CI: 1.08–2.40).

**Table 3:** Associations between ARS score and clinical outcomes.

**A**		**Fractures**					
** **	**ARS Score**	Yes n = 1,452 (%)	No n = 14,850 (%)	Unadjusted OR (95% CI)	p value	Adjusted OR (95% CI)	p value
** **	0	169 (11.6)	2,294 (15.4)	REF	-	REF	-
** **	1	360 (24.8)	3,920 (26.4)	1.25 (1.03–1.51)	0.02	1.15 (0.95–1.39)	0.16
** **	2–3	401 (27.6)	4,361 (29.4)	1.25 (1.04–1.50)	0.02	1.17 (0.97–1.42)	0.11
** **	4+	522 (36.0)	4,275 (28.8)	1.66 (1.38–1.99)	<0.01	1.56 (1.29–1.88)	<0.01
**B**		**Delirium**					
	**ARS Score**	Yes n = 362 (%)	No n = 15,940 (%)	OR (95% CI)	p value	AOR^a^ (95% CI)	p value
	0	33 (9.1)	2,430 (15.2)	REF	-	REF	-
	1	65 (18.0)	4,215 (26.4)	1.14 (0.75–1.73)	0.55	1.05 (0.69–1.61)	0.81
	2–3	149 (41.2)	4,613 (28.9)	2.38 (1.63–3.48)	<0.01	2.14 (1.46–3.15)	<0.01
	4+	115 (31.8)	4,682 (29.4)	1.81 (1.22–2.67)	<0.01	1.61 (1.08–2.40)	0.02

^a^Adjusted for age, sex, race, length of stay, Elixhauser comorbidity score, census region, urban/rural status, hospital size (number of beds), and hospital teaching status.

Abbreviations: ARS, anticholinergic risk scale; AOR, adjusted odds ratio; CI, confidence interval; OR, odds ratio REF; referent group.

### ED Visit and Inpatient Readmission

Estimates of associations between ARS score and 30-day ED visit and inpatient readmission are given in Table 4A and 4B, respectively. Unadjusted Cox results showed that individuals with the greatest ARS score (≥4) were significantly more likely to visit the ED within 30-days of inpatient discharge compared to those not prescribed medications with anticholinergic effects (hazard ratio (HR): 1.29, 95% CI: 1.08–1.54). Prior to covariate adjustment, no association between anticholinergic burden and 30-day inpatient readmission was observed. Hazard ratios slightly increased after covariate adjustment, showing that individuals with high to very high ARS scores were at significant risk of visiting the ED within 30 days of inpatient discharge compared to those without anticholinergic burden (ARS score 2–3: adjusted hazard ratio (AHR): 1.22, 95% CI: 1.02–1.46; 4+: AHR: 1.32, 95% CI: 1.10–1.58). Similarly, compared to individuals not prescribed anticholinergic medications (ARS score = 0), individuals with the greatest anticholinergic burden (ARS score ≥4) had a 16% greater risk of being readmitted to an inpatient setting within 30 days of inpatient discharge (AHR: 1.16, 95% CI: 1.01–1.33).

**Table 4:** Associations between ARS score and healthcare utilization outcomes.

**A**		**30-Day ED Visit**				
	**ARS Score**	Visits/patients (%)	HR (95% CI)	p value	AHR^a^ (95% CI)	p value
	0	171/2,463 (6.9)	REF	-	REF	-
	1	300/4,280 (7.0)	1.01 (0.84–1.22)	0.89	1.04 (0.86–1.26)	0.66
	2–3	390/4,762 (8.2)	1.19 (1.00–1.43)	0.05	1.22 (1.02–1.46)	0.03
	4+	423/4,797 (8.8)	1.29 (1.08–1.54)	0.01	1.32 (1.10–1.58)	<0.01
**B**		**30-Day Readmission**				
	**ARS Score**	Readmissions/patients (%)	HR (95% CI)	p value	AHR^a^ (95% CI)	p value
	0	312/2,463 (12.7)	REF	-	REF	-
	1	545/4,280 (12.7)	1.01 (0.88–1.16)	0.92	1.04 (0.90–1.19)	0.60
	2–3	652/4,762 (13.7)	1.09 (0.95–1.25)	0.21	1.12 (0.98–1.29)	0.10
	4+	671/4,797 (14.0)	1.11 (0.97–1.27)	0.11	1.16 (1.01–1.33)	0.04

^a^Adjusted for age, sex, race, length of stay, Elixhauser comorbidity score, census region, urban/rural status, hospital size (number of beds), and hospital teaching status.

Abbreviations: ARS, anticholinergic risk scale; AHR, adjusted hazard ratio; CI, confidence interval; ED, emergency department; HR, hazard ratio; REF, referent group.

## Discussion

Many cardiac, gastrointestinal, allergy and psychiatric medications exhibit anticholinergic potential. Multiple studies have demonstrated risks associated with anticholinergic burden in the older adult population, including falls, fractures, cognitive impairment, pneumonia, and hospital readmission [4–12, 23]. Individuals with PD may be susceptible to anticholinergic effects due to cholinergic dysfunction as part of the disease process, and the exposure to anticholinergic substances in the form of antiparkinson medications. Overall, there is need for more studies to examine anticholinergic effects in PD, both among PD populations with varying exposure to anticholinergic medications and between PD and non-PD populations with comparable exposures to these medications. Such studies will be essential to informing future best practice guidelines, as well as public health policies. Using data from a large cohort of more than 16,000 individuals with PD admitted to hospital between 2000 and 2011, we examined anticholinergic use and the extent to which anticholinergic burden was associated with adverse clinical and health service utilization outcomes.

Despite known risks of prescribing medications with anticholinergic effects to older adults, estimates suggest that over one-third of medications prescribed to the elderly have anticholinergic properties [24]. Our findings show that even after excluding PD medications, anticholinergic medications were prescribed to more than half of our PD inpatient cohort, which is consistent with a prior report that anticholinergic burden in PD is largely attributed to the use of non-PD medications [25]. We also found that patients with the highest anticholinergic burden were more likely to be diagnosed with a fracture and delirium compared to those not taking medications with anticholinergic effects. Previous studies have demonstrated that traumatic injuries are prevalent and a leading cause of morbidity, mortality, and disability in PD. One study found that a PD diagnosis was four times more common in a sample of 1,066,404 hospitalizations for acute hip fractures (age- sex-adjusted prevalence ratio 4.02, 4.00–4.03) than in the general population [26]. Post hip fracture mortality is increased in Medicare beneficiaries diagnosed with PD compared to the general Medicare population (AHR 2.41, 2.37–2.46) [27]. Hip fracture is also an independent predictor of nursing home residence (AOR 2.10, 2.04–2.15) [27–30].

In PD, as in the general older adult population, acute metabolic or infection insults can precipitate delirium. Multiple studies have found an association between anticholinergic medication use and delirium [31–33], resulting in prolonged hospitalization and rehabilitation [34]. However, in PD, acute delirium may lead to loss of motor function. A recent study of 80 individuals with PD examined risk factors for persistent significant motor deterioration (e.g. going from walking independently to requiring an assist device) after an acute inflammatory process (such as a respiratory tract infection). Individuals with PD who had delirium were 15 times more likely to have persistent PD motor disability six months after the acute illness resolved (AOR 15.89, 3.23–78.14) [35]. Both cross-sectional and longitudinal studies suggest that cognitive impairment is the most commonly observed non-motor feature of PD; however, the determinants of cognitive dysfunction, particularly early in disease, are not clear. An international, multi-site study of 423 individuals with PD recently reported that approximately 10% of newly diagnosed cases of PD had measurable cognitive impairment at disease presentation [36]. Most recently, a large cohort study examined anticholinergic burden in PD and found that there were no differences in global cognition or assessments of attention, memory, and executive function at 1.5 years in groups of users and non-user of anticholinergic medications [37]. This is in contrast to other studies that found executive dysfunction and attentional deficits in individuals with PD exposed to anticholinergic medications, even in subclinical doses [38], and in contrast to multiple studies that relate anticholinergic drug exposure to incident dementia [39–41]. Our data raise important questions about the extent to which traumatic injuries, motor decline, and cognitive dysfunction are preventable in PD. The potential public health impact of these initial data, if confirmed, is substantial.

Our findings that PD inpatients with high anticholinergic burden were significantly more likely than those not treated with anticholinergic acting medications to visit the ED and be readmitted within 30 days of discharge are congruent with prior reports of adverse events in other elderly populations [7, 11–13]. Non-PD medications with anticholinergic effects may often be substituted for equally effective non-anticholinergic agents: a portion of the ED visits and inpatient readmissions we observed are thus potentially preventable. If replicated using other data sources, our findings may serve to inform care center policies and practices pertaining to prescribing, adverse event reporting, and reimbursement.

Although there are published lists of drugs that should be avoided by elderly populations (such as Beers Criteria [42]), inappropriate prescribing stills occurs and is a contributor to preventable adverse health outcomes [2, 43, 44]. Many care centers have implemented computerized provider order entry systems to improve the quality of care while reducing variable operating costs. These systems are designed to leverage patient data and pre-programmed drug information to warn clinicians if ordered medications are potentially contraindicated on a case-by-case basis [45]. Prompted warnings based on anticholinergic burden may prove beneficial in PD, as this would allow care providers who may otherwise prescribe anticholinergic acting medications to reevaluate their decisions and make medication substitutions where appropriate. However, current warning systems for antidopaminergic medication use in PD have not always produced adequate physician response [46]. In instances where individuals are diagnosed with outcomes believed to result from anticholinergic burden, mandatory in-hospital reporting and medication reconciliation may be necessary to improve future quality of care.

Our study has several strengths. Study data originated from multiple care centers in the United States and include information on individuals with PD from multiple payers. Academic centers were overrepresented in our dataset and are more likely to have providers with PD expertise available on-site. In-depth data for each index encounter enabled us to adjust multivariable models for *a priori* defined covariates that may modify or confound the association between anticholinergic burden and adverse outcomes in PD. We accounted for differences in health status across compared groups by including a weighted comorbidity summary score in our multivariable logistic regression and Cox proportional hazard models, which has shown to be statistically superior compared to adjustment for individual comorbidity counts [47]. Finally, our results are congruous with previous studies of other older adult populations.

In spite of these strengths, limitations in our study design should be considered when interpreting these initial data. Confounding by indication or protopathic bias may affect our risk estimates, as we did not have information on PD severity or access to outpatient prescription history, nor were we able to perform time-lag analyses. Although comorbidity summary scores may effectively summarize health status and predict in-hospital mortality [20, 47], it is possible that differences in factors that could not be accounted for by our study, including outpatient use of prescribed or over-the-counter central nervous system acting medications not documented upon admission, contributed to individuals with the greatest anticholinergic burden returning to hospital at a higher rate than those with no anticholinergic burden. Moreover, we did not have any validation data available to perform external adjustment to reduce possible residual confounding bias [48]. It is possible that antidepressants and hypnotics with anticholinergic potential were prescribed for early symptoms of dementia or palliative treatment of advanced PD, conditions that independently predict admission to hospital for falls and altered mental status [49, 50]. Additionally, we could not account for significant medication changes post inpatient discharge that could impact anticholinergic burden, nor measure ED visits or readmissions to care centers that were not subscribed to Health Facts®, which could lead to possible over or underestimation of reported risks. Our choice to use the ARS, a popular anticholinergic burden measurement tool that has been validated in other United States health databases, may be responsible in part for observed associations between anticholinergic burden and clinical and healthcare utilization outcomes. Currently, there are many distinct drug lists used to measure anticholinergic burden, with considerable disagreement among developed scales [51–55]. Anticholinergic measurement tools should not be used in settings that dramatically differ from those in which the scales were developed, as differences in drug availability may adversely impact measured anticholinergic exposure [51, 56]. A recent systematic review on the use of anticholinergic scales found that cumulative exposure to anticholinergic medications measured using the ARS was associated with cognitive and functional disorders [57]. Furthermore, a New Zealand-based study of older adults examined weather nine published anticholinergic burden scales, including the ARS, were associated with adverse health outcomes [13]. Investigators found that scores derived from all nine scales were independently associated with an increased risk of hospital admission, including admission for falls [13]. To date, no scale has demonstrated a clear relationship with mortality [57]. Although our findings are supported by some studies that used the ARS to investigate associations between anticholinergic medication exposure and the diagnosis of adverse outcomes in older adult populations [13, 18, 58, 59], our study is the first to use the ARS to examine these associations among individuals with PD. Future work is needed to examine how differences in anticholinergic burden measurement impact the predictive validity of clinically relevant outcomes in Cerner Health Facts® and other health databases, both in older adult populations and in subpopulations that may be most sensitive to anticholinergic effects.

It is important to note that medications included in the ARS, such as haloperidol and metoclopramide, possess central antidopaminergic activity in addition to anticholinergic properties and that these medications are independent predictors of adverse outcomes in inpatients with PD [60, 61]. There is also growing evidence that exposure to the most potent dopamine receptor blocking agents is associated with increased risk of mortality, both in the general population and among individuals with PD [62–64]. Since there are no widely accepted standards for assessing dopamine receptor blocking activity of medication included in the ARS, we were unable to account for differences in antidopaminergic activity, if any, within compared groups in our study. Future studies that examine how prescribed medications with both anticholinergic and antidopaminergic properties may contribute to adverse events in older adult populations, especially those with PD, are required.

Although medications dispensed in inpatient settings were presumed continuations of outpatient treatment, lacking outpatient pharmacy data in Health Facts® limited our ability to ascertain whether anticholinergic burden was temporally associated with examined clinical outcomes. This is particularly true for anticholinergic burden and the diagnosis of delirium, since haloperidol and quetiapine may have been prescribed for acute changes in the mental status [65, 66]. To better understand possible confounding by indication in this context, future studies that compare use of a particular drug for the same indication among individuals with PD and varying degrees of anticholinergic burden are needed. Finally, we did not make adjustments for multiple comparisons, as our analyses are exploratory. Despite study limitations, prior reports of adverse outcomes with anticholinergic burden in older adults support an apparent association between ARS medications use and the diagnosis of adverse clinical and health service utilization outcomes in PD [4–7].

In conclusion, we found a positive association between anticholinergic burden and adverse clinical (fracture and delirium) and health service (30-day ED visit and inpatient readmission) outcomes in a large cohort of inpatients with PD. Anticholinergic burden was primarily attributed to the use of non-PD medications. Although study replication is warranted, initial findings suggest that older adults with PD may benefit from limited use of non-PD medications with anticholinergic effects.
